# Anaplastic Large Cell Lymphoma (ALCL) With a Sarcomatoid Variant Presenting As Distributive Shock in a 41-Year-Old Female: A Case Report

**DOI:** 10.7759/cureus.55235

**Published:** 2024-02-29

**Authors:** Leena Alhusari, Mahmoud Abdallah, Bassel Dakkak, Taysir Bsiso, Muhammad Jamil

**Affiliations:** 1 Internal Medicine, Marshall University Joan C. Edwards School of Medicine, Huntington, USA; 2 Hematology and Medical Oncology, Marshall University Joan C. Edwards School of Medicine, Huntington, USA

**Keywords:** sarcomatoid variant, anaplastic large cell lymphoma (alcl), distributive shock, alk-positive, anaplastic lymphoma kinase

## Abstract

The sarcomatoid variant is considered a rare subtype of anaplastic large cell lymphoma. We present a 40-year-old diabetic female who was evaluated in the ER for distributive shock, requiring vasopressors and mechanical ventilation. An extensive workup was negative for infection. A serial CT scan of the abdomen and pelvis showed evolving lymphadenopathy, and a biopsy revealed malignant anaplastic lymphoma cells with a sarcomatous variant. The oncology team recommended the initiation of inpatient chemotherapy; however, the family opted to proceed with comfort care measures.

## Introduction

Sarcomatoid anaplastic large cell lymphoma (ALCL) is a rare histologic variant of ALCL resembling a soft tissue sarcoma with its spindle-shaped bizarre cells [1]. Those cells are defined by the expression of CD-30 receptors, which defines them as lymphoid cells. Around 25-60% of cases express the unique anaplastic lymphoma kinase (ALK) enzyme that actively aids in the proliferation of malignant lymphoma cells [2].

The production of cytokines and the expression of their receptors can be found in several malignancies, including malignant lymphoma [3]. Those cytokines play a vital role in manifesting the clinical signs and symptoms of the underlying malignancy. The elevated serum concentration of these cytokines results in reducing the vascular tone and ultimately presenting in refractory shock [4]. We present a case of sarcomatous ALCL presenting with distributive shock.

## Case presentation

Our patient is a 40-year-old diabetic female who presented to the ER with progressively worsening nasal congestion, shortness of breath, generalized body weakness, nausea, and diarrhea for five days. The patient was initially evaluated at an outside facility, tested positive for enterovirus and rhinovirus, and treated conservatively for viral illness. However, her symptoms kept worsening.

Upon initial evaluation, she was afebrile and in shock with hypotension and tachycardia. She had labored breathing, her chest was clear for auscultation, and otherwise, the examination was unremarkable. Her mental status worsened, and the patient was intubated and required mechanical ventilation. Her blood pressure remained low despite initial resuscitation and the need for vasopressors. She was admitted to the ICU for distributive shock and acute hypoxic respiratory failure.

Her initial blood work was evident for leukocytosis, acute kidney injury, lactic acidosis, and mildly elevated liver enzymes (Table 1). The patient had normal, random serum cortisol levels. A CT chest revealed bibasilar atelectasis and pneumonia-like changes, which started on broad-spectrum antibiotics. The CT abdomen and pelvis were initially negative.

**Table 1:** Blood work results

Test	Lab result	Reference range
White cell count	19.77	4.5-10 × 10^9/L
Hemoglobin	12.5	11-18 × 10^9/L
Platelets	138	150-440 × 10^9/L
Blood urea nitrogen (BUN)	21	5-18 × 10^9/L
Creatinine	3.4	0.7-1.4 × 10^9/L
Lactic acid	5.0	0.7-2.0 mmol/L
Aspartate aminotransferase (AST)	199	15-37 unit/L
Alanine transaminase (ALT)	83	12-78 unit/L
Alkaline phosphatase (ALP)	8.2	45- 117 unit/L

During the ICU stay, the patient developed worsening leukocytosis and transaminitis, for which the gastroenterology team was consulted, and endoscopic retrograde cholangiopancreatography was performed, which showed benign stenosis of the ampulla of Vater requiring sphincterotomy and stenting. The patient’s kidney function kept worsening, and he was started on continuous renal replacement therapy. The laboratory results were not suggestive of tumor lysis syndrome, given normal levels of serum phosphate, uric acid, and potassium.

**Figure 1:**
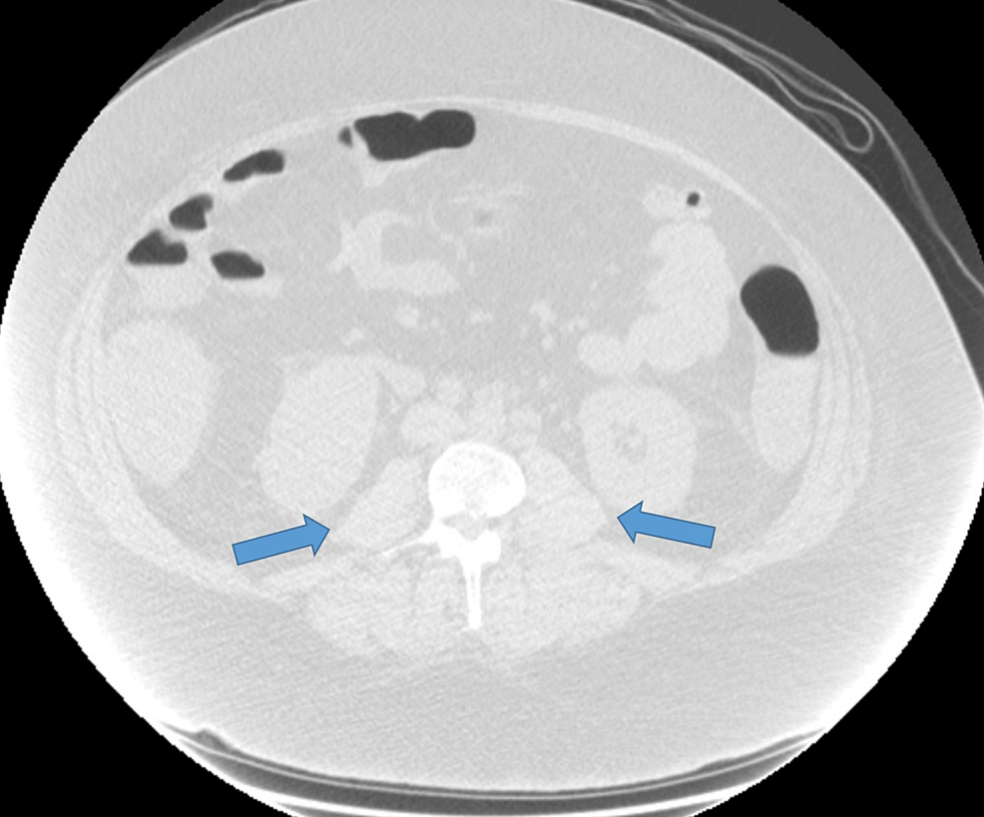
CT abdomen and pelvis showing periportal and mild retroperitoneal lymphadenopathy (blue arrows) CT: computed tomography

The patient had an increasing vasopressor requirement, worsening leukocytosis, and raised inflammatory markers, including C-reactive protein and procalcitonin, suggestive of infection warranting infectious disease. Consult for fungal workups, including opportunistic organisms like histoplasma, cryptocccus, toxoplasma, and aspergillus, that came back negative. Sputum and urine cultures were all negative. Flow cytometry of the peripheral blood smear showed a small, atypical T-cell population (3% of total events) with high side scatter. Repeat CT abdomen and pelvis noted intrabdominal and retroperitoneal lymphadenopathy (Figure 1).

A CT-guided pelvic lymph node biopsy was performed, which later revealed anaplastic large B-cell lymphoma with a sarcomatous variant positive for ALK. The patient was started on steroids, and antibiotic coverage narrowed as refractory hypotension was thought likely to be related to lymphoma.

The oncology team recommended the initiation of inpatient chemotherapy as a last resort. Patient pressure continued to increase, and the patient had minimal ability to come off ventilation, for which the family decided to proceed with comfort care measures before any systemic treatment for ALCL was started. The patient passed away shortly afterward.

## Discussion

The large, spindle-shaped, bizarre neoplastic cells are characteristic of a sarcomatous variant of ALCL. This histologic subtype of ALCL is considered one of the rarest variants of ALCL. The pathognomonic histologic features consist of large lymphoid cell proliferation with strong expression of the cytokine receptor CD30 [5]. It has been reported that 25-60% of ALCL carry the t(2;5)(p23;q35) translocation that results in the production of the novel chimeric protein ALK [2]. ALCL frequently involves lymph nodes and occasionally involves extra-nodal sites, such as the skin, soft tissues, bone, bone marrow, liver, lungs, and gastrointestinal tract [6]. The sarcomatoid variant of anaplastic large B-cell lymphoma typically presents with B symptoms and lymphadenopathy or cutaneous lesions [7]. Less common presentations have been reported in the literature, such as cases imitating breast cancer or bladder tumors [8]. However, an extremely rare presentation is a distributive shock in these patients, possibly related to tumor cytokine production. Similar clinical manifestations have been reported in other case reports and clinical studies that prompted extensive but mostly negative microbiology and serology tests for a presumed infection and/or sepsis [9].

Our case discussed above illustrates the complexity of diagnosis, with initial symptoms of respiratory distress and hypotension necessitating a higher level of care and ICU admission. The patient’s deteriorating condition and poor response to supportive treatment led to further investigations, like CT scans, which showed evidence of lymphadenopathy and the diagnosis of sarcomatoid ALBL, confirmed through a pelvic lymph node biopsy. The only diagnostic clue was a worsening clinical course despite prompt management in the background of distributive shock.

Anthracycline-based regimens are usually the treatment option for patients with ALK-positive ALCL. Those regimens include the combination of cyclophosphamide, doxorubicin, vincristine, and prednisolone (CHOP) or CHOEP (CHOP plus etoposide), which provide a favorable prognosis, except in patients with multiple International Prognostic Index factors [10]. Of note, brentuximab vedotin is the first monoclonal antibody approved for treating ACLC, ending a decades-long wait for a targeted approach.

## Conclusions

Our manuscript describes a case of sarcomatous ALCL presenting with distributive shock. The suggested mechanism for ALCL-induced shock is the increased cytokine production by malignant CD-30-positive cells, which induces vasodilatation. In summary, this case emphasizes the need for a comprehensive, multidisciplinary approach in evaluating patients with atypical and severe presentations, especially when initial treatments fail to yield improvement. Collaboration between infectious disease specialists, hematology/oncology teams, radiologists, and pathologists is crucial to achieving a timely and accurate diagnosis. It also underscores the importance of early recognition and aggressive management in such a scenario.

## References

[1] Yu L, Yan LL, Yang SJ (2014). Sarcomatoid variant of ALK- anaplastic large cell lymphoma involving multiple lymph nodes and both lungs with production of proinflammatory cytokines: report of a case and review of literature. Int J Clin Exp Pathol.

[2] Al-Hashmi I, Decoteau J, Gruss HJ (2001). Establishment of a cytokine-producing anaplastic large-cell lymphoma cell line containing the t(2;5) translocation: potential role of cytokines in clinical manifestations. Leuk Lymphoma.

[3] Nomura M, Narita Y, Miyakita Y (2013). Clinical presentation of anaplastic large-cell lymphoma in the central nervous system. Mol Clin Oncol.

[4] Shimamoto T, Hayashi S, Ando K (2001). Anaplastic large-cell lymphoma which showed severe inflammatory status and myelodysplasia with increased VEGF and IL-6 serum levels after long-term immunosuppressive therapy. Am J Hematol.

[5] Stein H, Foss HD, Dürkop H (2000). CD30(+) anaplastic large cell lymphoma: a review of its histopathologic, genetic, and clinical features. Blood.

[6] Hsu SM, Waldron Jr JW, Hsu PL, Hough AJ Jr (19931). Cytokines in malignant lymphomas: review and prospective evaluation. Human pathology.

[7] Pereira EM, Maeda SA, Reis-Filho JS (2002). Sarcomatoid variant of anaplastic large cell lymphoma mimicking a primary breast cancer: a challenging diagnosis. Arch Pathol Lab Med.

[8] Allory Y, Merabet Z, Copie-Bergman C, Lange F, Yiou R, Gaulard P (2005). Sarcomatoid variant of anaplastic large cell lymphoma mimics ALK-1-positive inflammatory myofibroblastic tumor in bladder. Am J Surg Pathol.

[9] Tsuyama N, Sakamoto K, Sakata S, Dobashi A, Takeuchi K (2017). Anaplastic large cell lymphoma: pathology, genetics, and clinical aspects. J Clin Exp Hematop.

[10] Laurent C, Do C, Gascoyne RD (2009). Anaplastic lymphoma kinase-positive diffuse large B-cell lymphoma: a rare clinicopathologic entity with poor prognosis. J Clin Oncol.

